# Supplementary material for: Ethnic Identity Profiles Among Adolescents in the ABCD Study: Associations with Resting State Functional Connectivity and Perceived Discrimination
Source: bioRxiv. 2025 Aug 28:2025.08.24.671805. Preprint. [Version 1] doi: 10.1101/2025.08.24.671805 (PMC12407776; doi:10.1101/2025.08.24.671805)
Supplement: Supplement 1 [file media-1.docx]

**Supplemental Information for**

**Ethnic Identity Profiles Among Adolescents in the ABCD Study: Associations with Resting State Functional Connectivity and Perceived Discrimination**

**Taylor R. Jancetic^1^, Micaela Lembo^3^, Chloe Hampson^2^, Donisha D. Smith^1^, Julio A. Peraza^2^, Erin Thompson^1^, Mariana Sanchez^4^, Raul Gonzalez^1^, Alan Meca^5^, Angela R. Laird^2^**

^1^Department of Psychology, Florida International University, Miami, FL, USA
^2^ Department of Physics, Florida International University, Miami, FL, USA

^3^ Department of Epidemiology, Florida International University, Miami, FL, USA

^4^ Department of Health Promotion and Disease Prevention, Florida International University, Miami, FL, USA

^5^ Department of Psychology, University of Texas-San Antonio, San Antonio, TX, USA

# **Supplemental Results**

## **Participants**


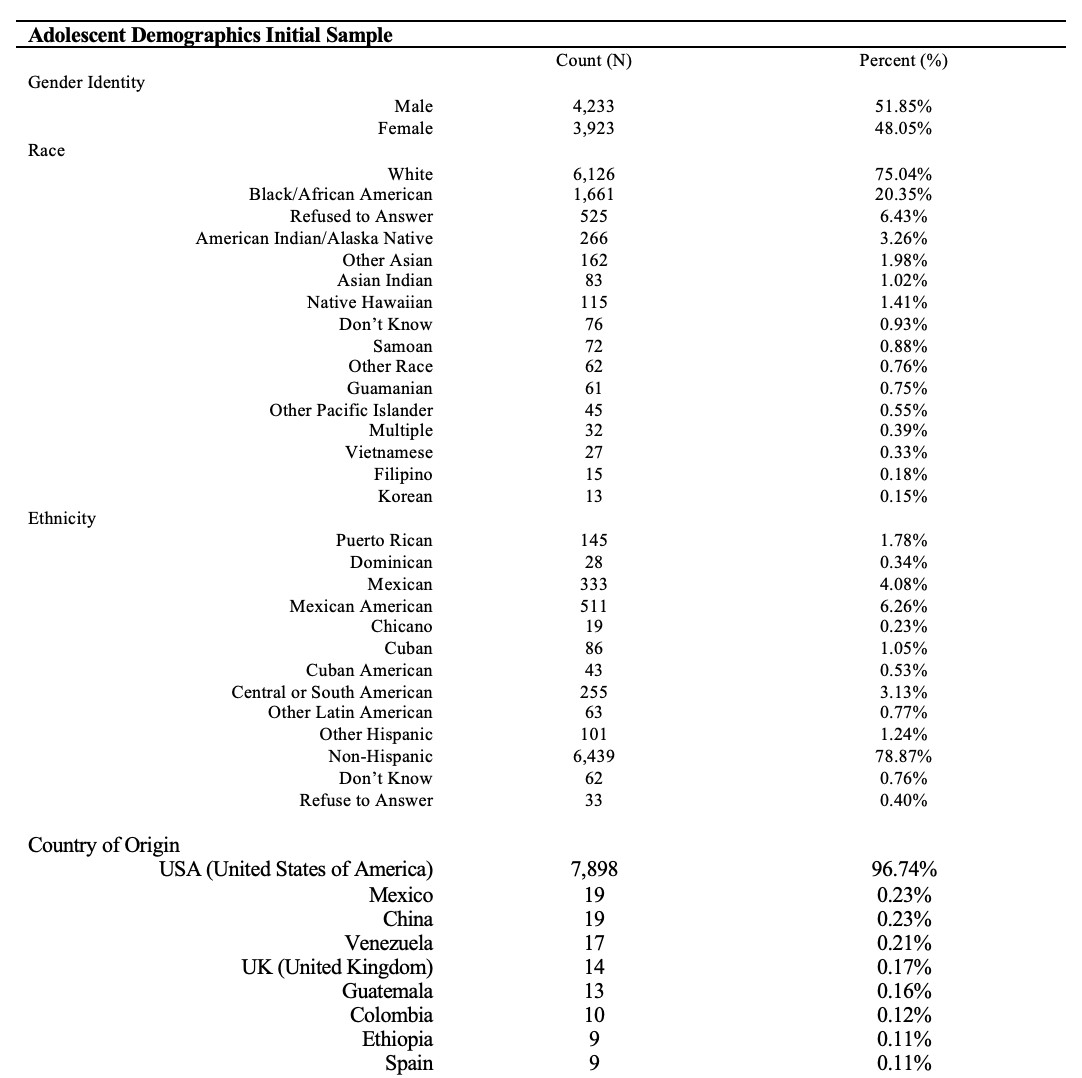


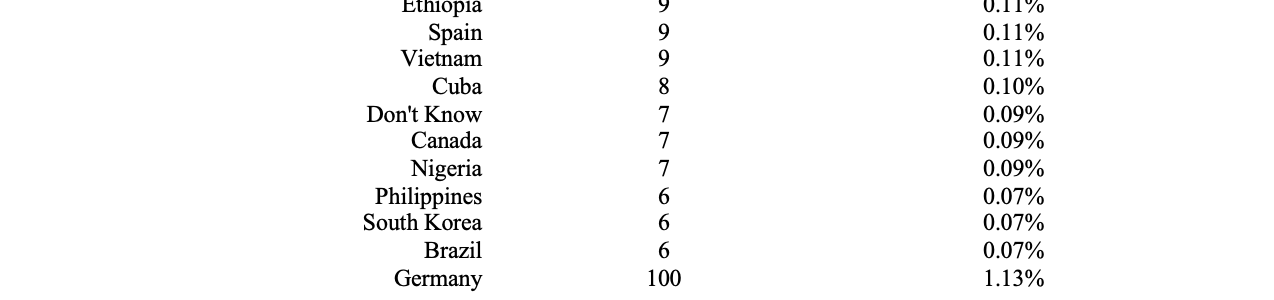


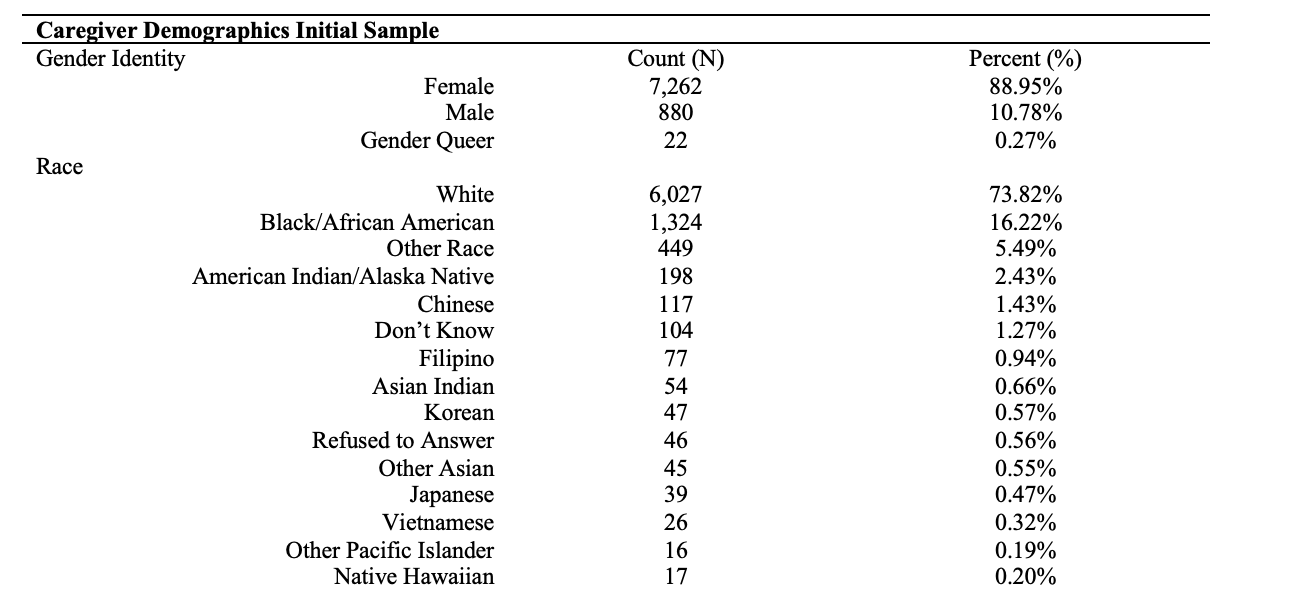


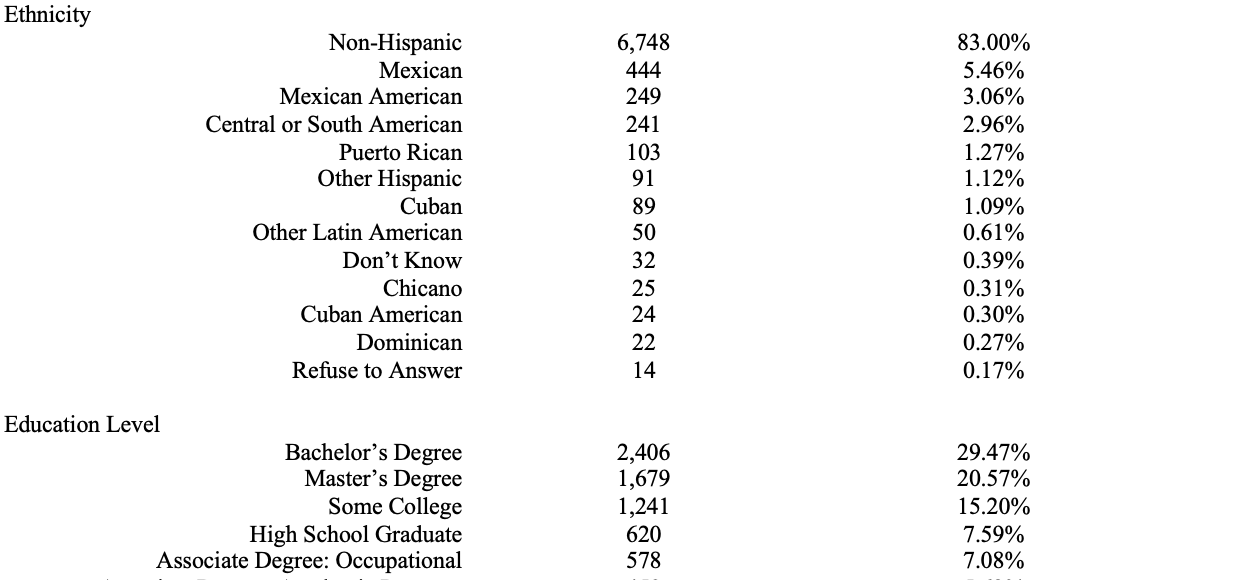


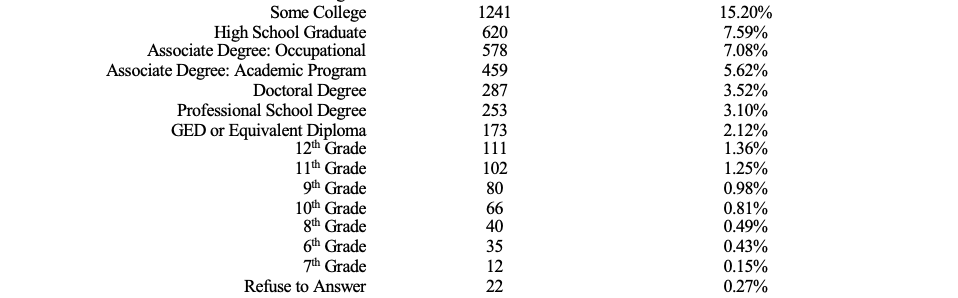


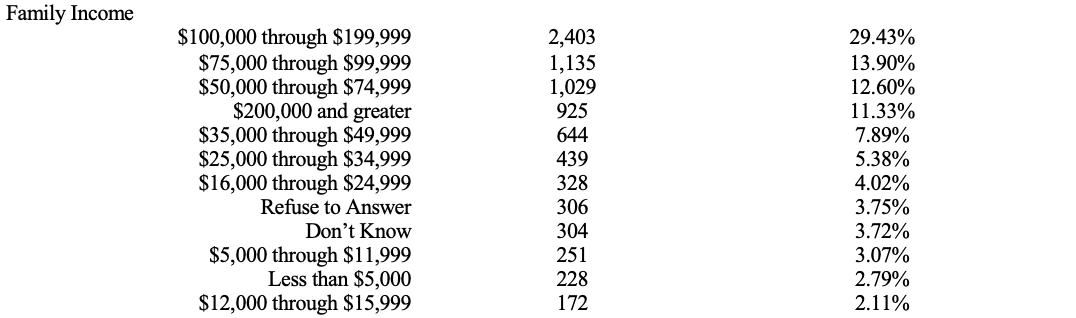


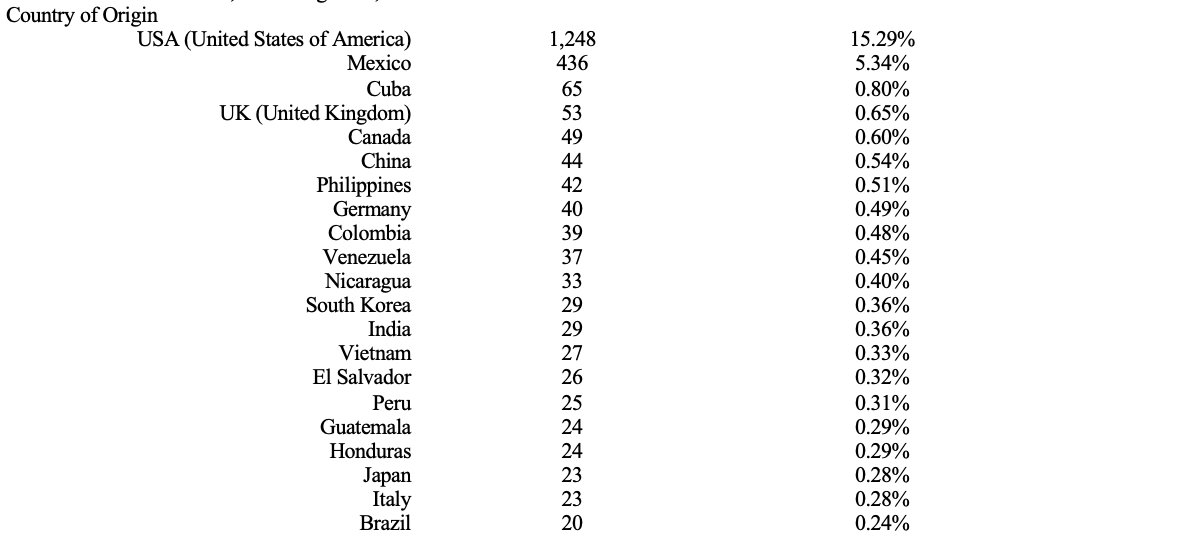


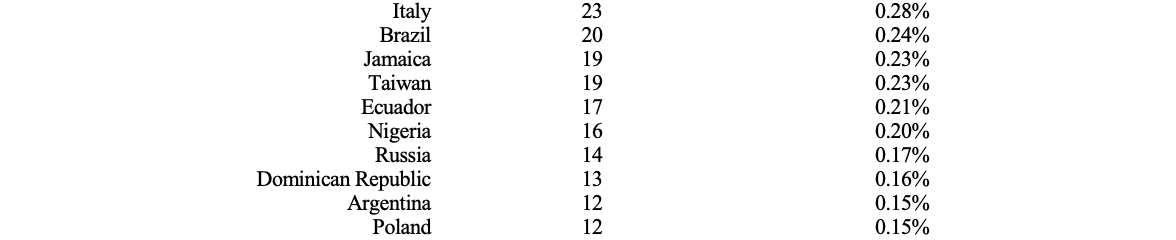


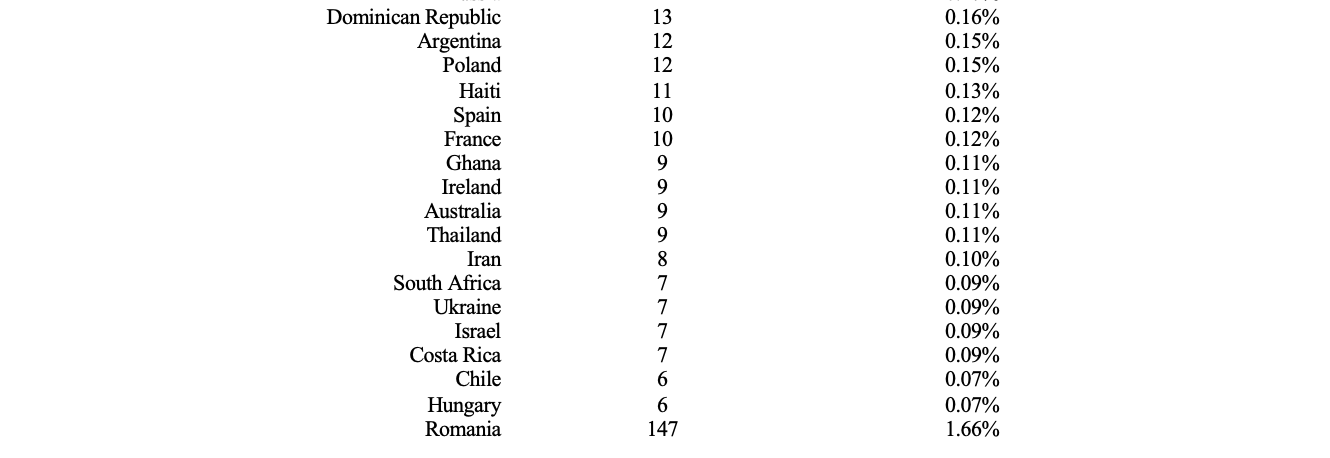


**Table S1. Full Demographics of MEIM-R Y3 Sample.** Variables which contained less than or equal to 5 participants were collapsed into reported categories to maintain anonymity [(CPRD, 2024)](https://www.zotero.org/google-docs/?w1OYik).

## **Resting State Functional Connectivity**
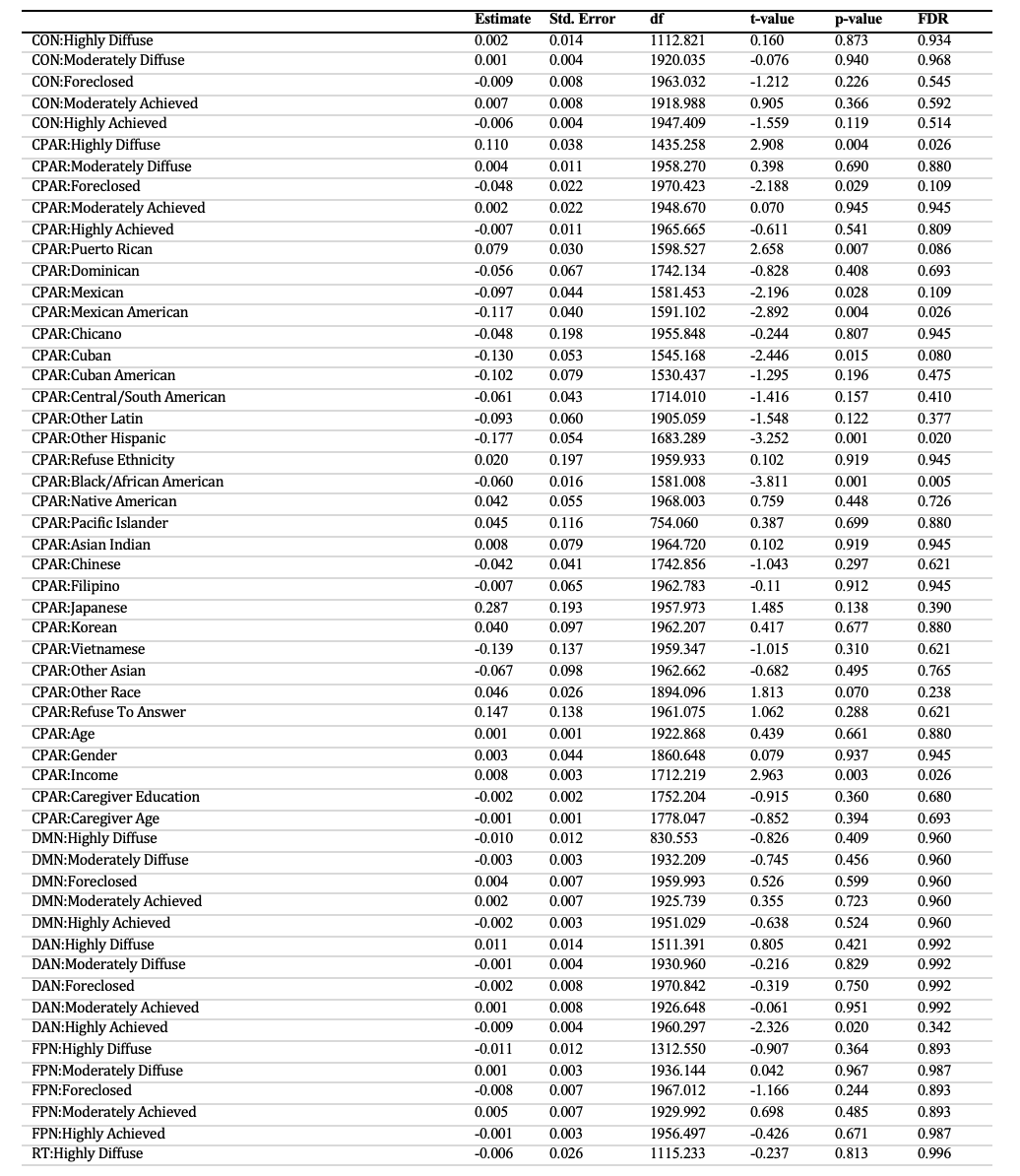


**
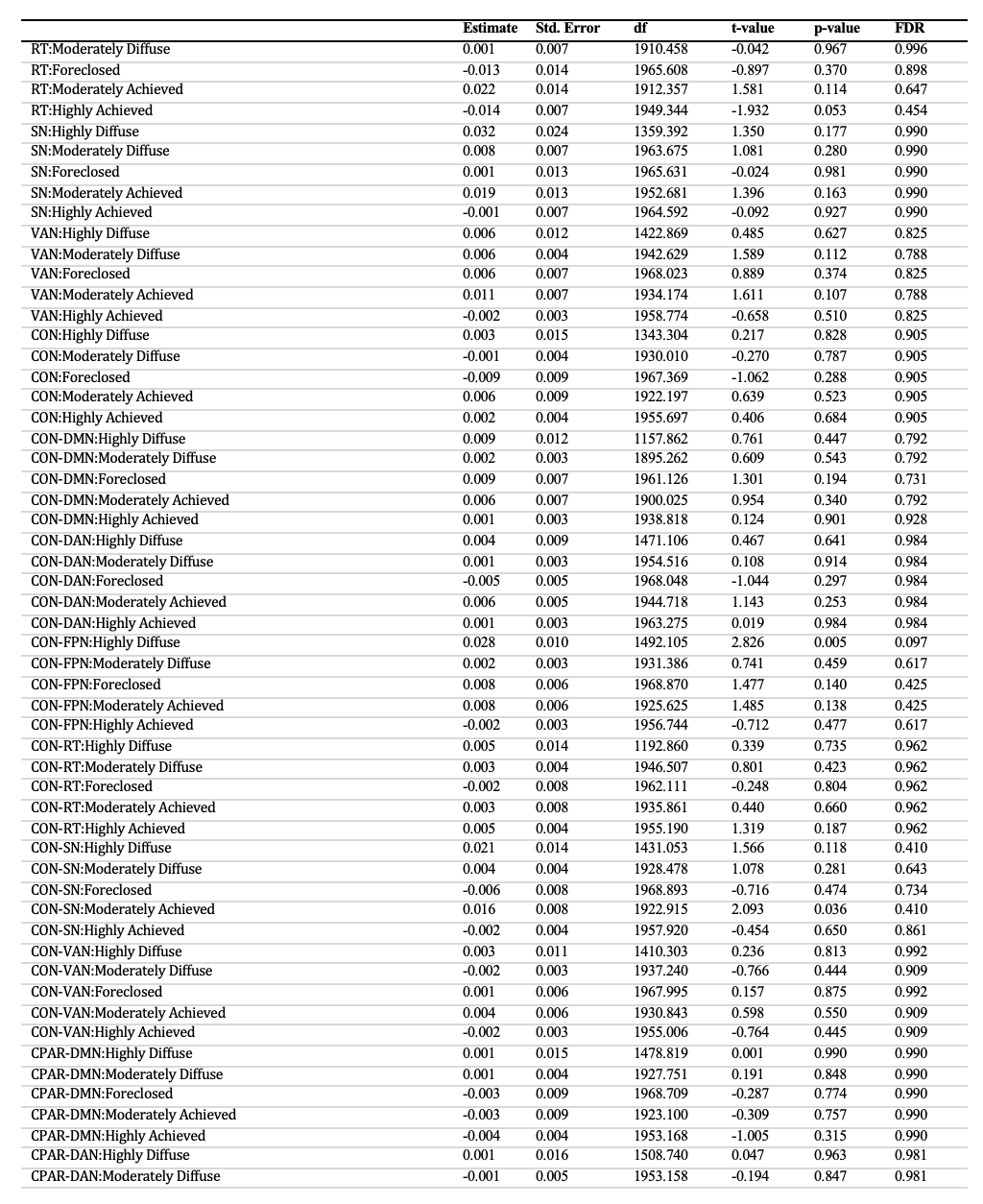
**


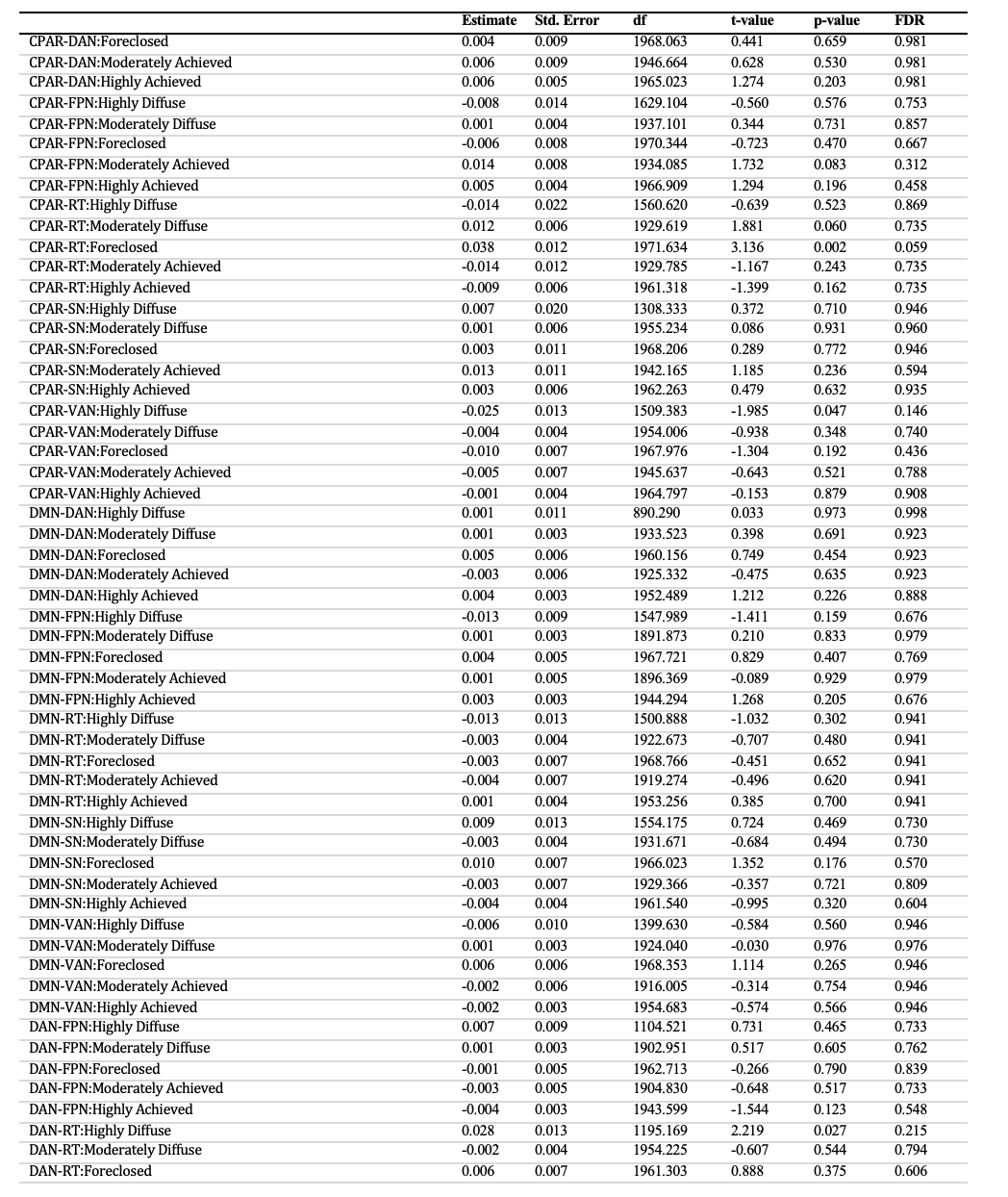


**
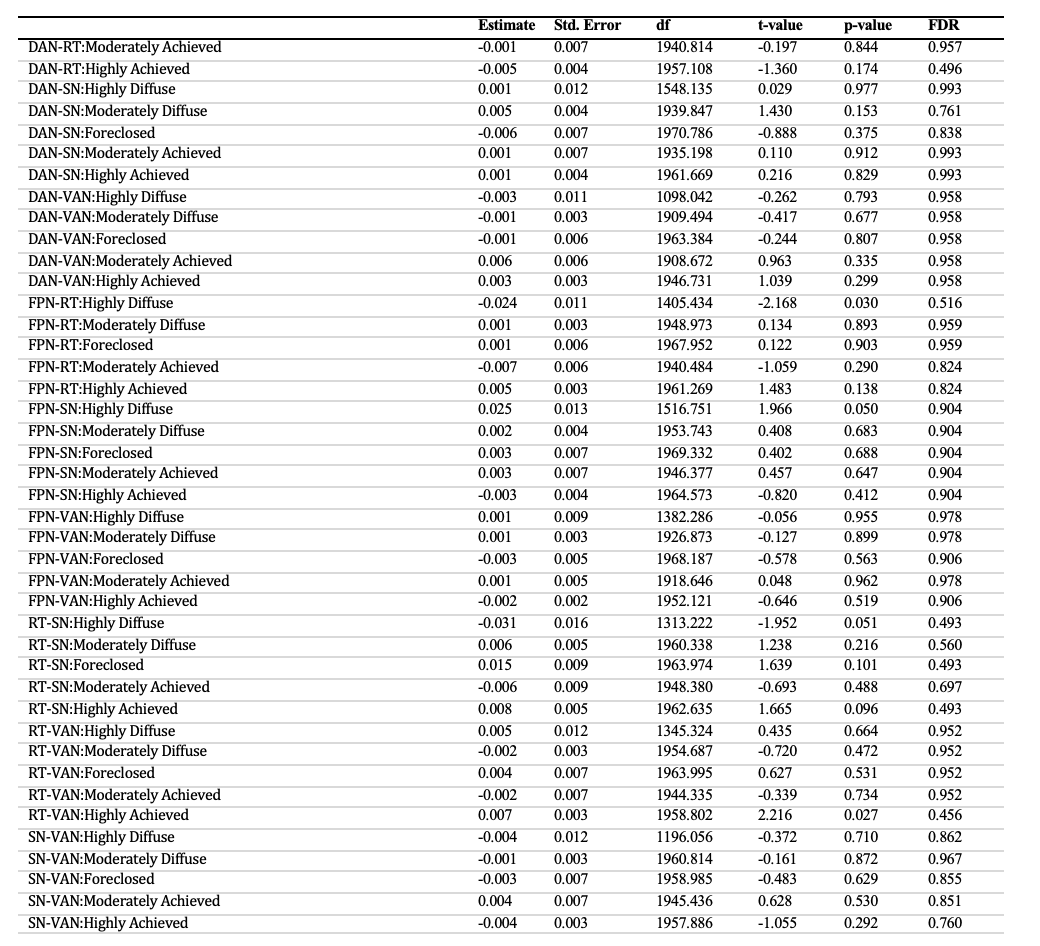
**

**Table S2. Resting State Functional Connectivity Results.** Statistical significance was determined by a p_FDR_–value of 0.05 or less. Degrees of freedom are denoted as df, standard error is denoted as Std. Error, and false discovery rate is denoted as FDR.

## **Moderation of Perceived Discrimination**

**
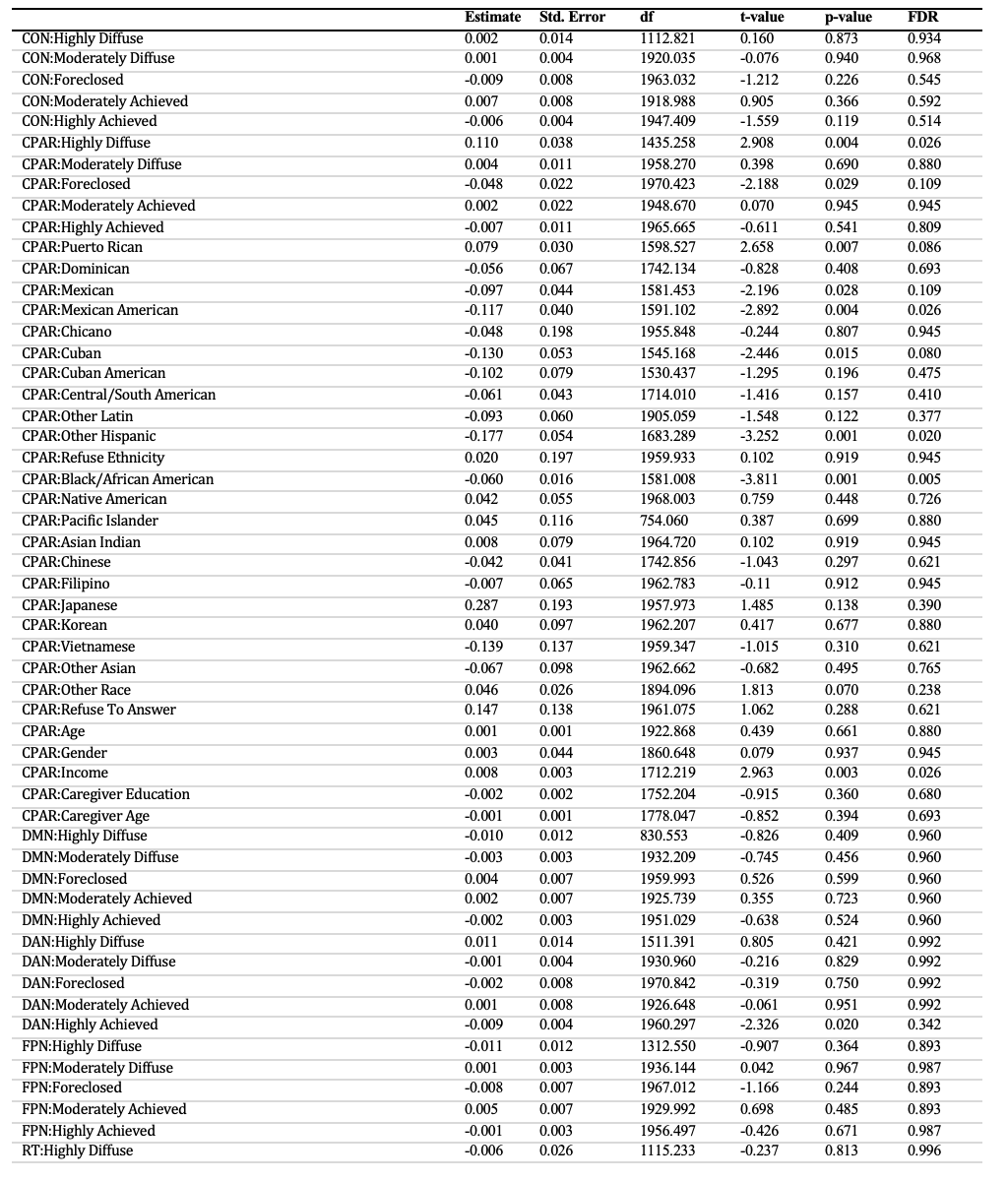
**

**
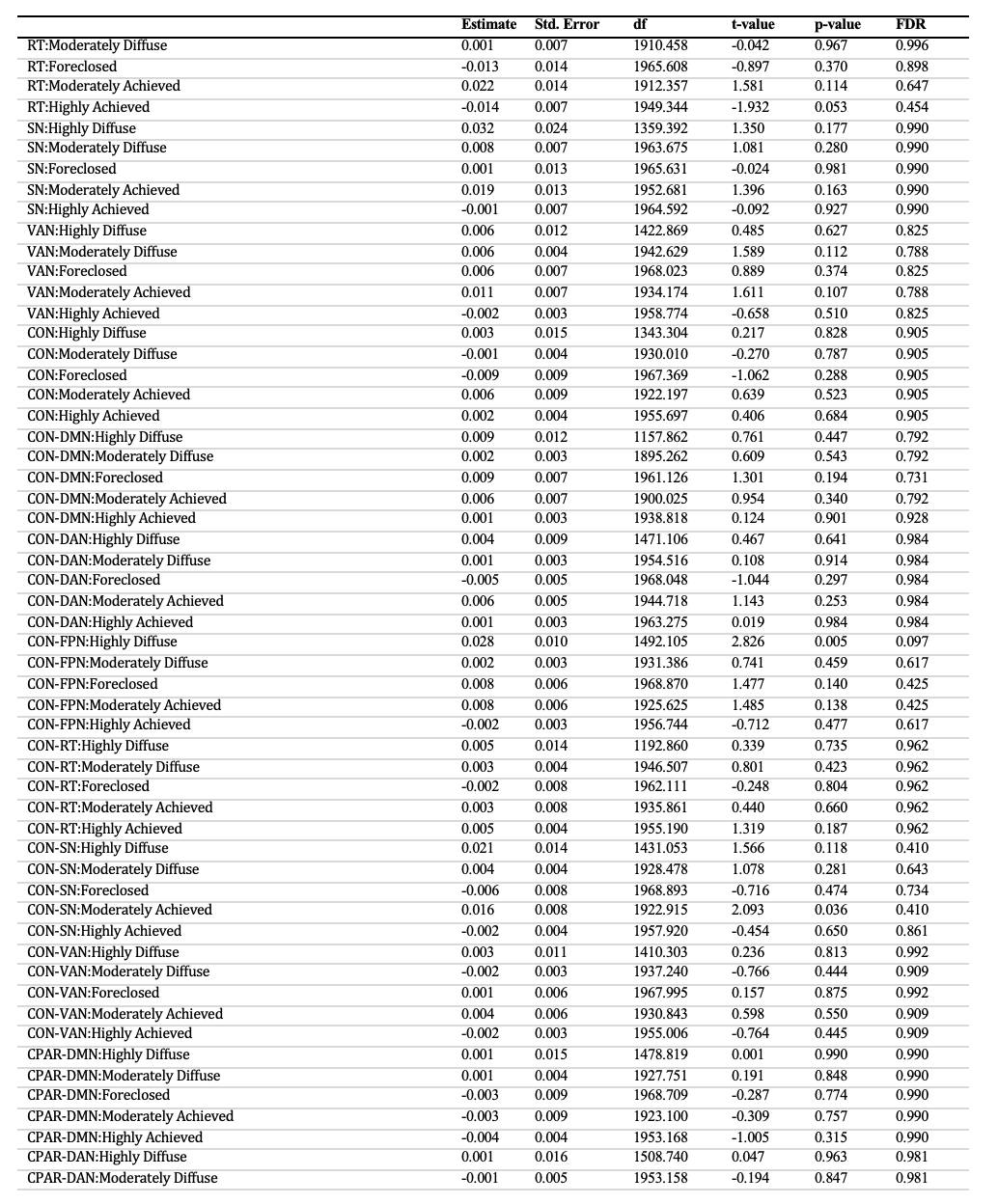
**

**
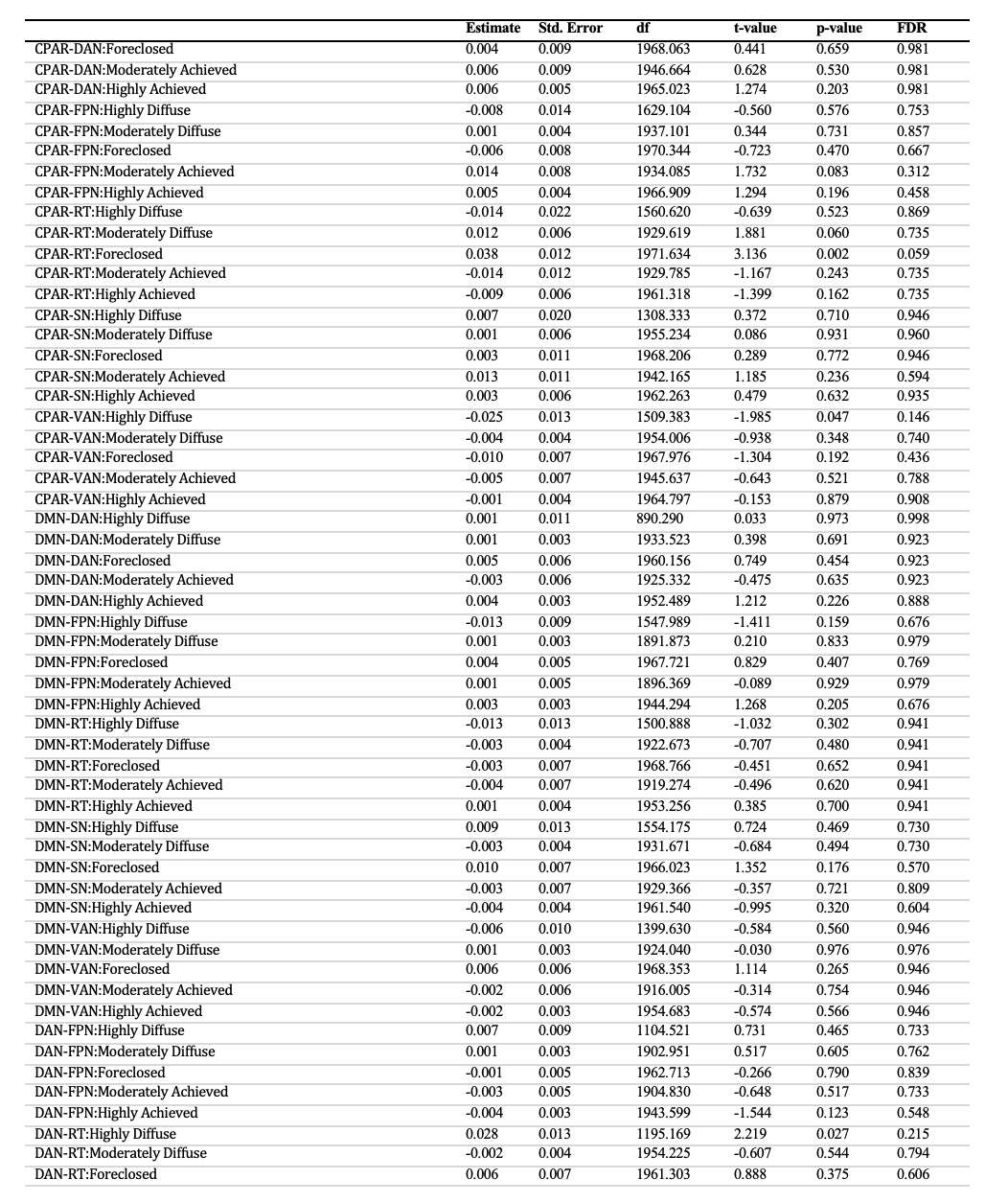
**

**
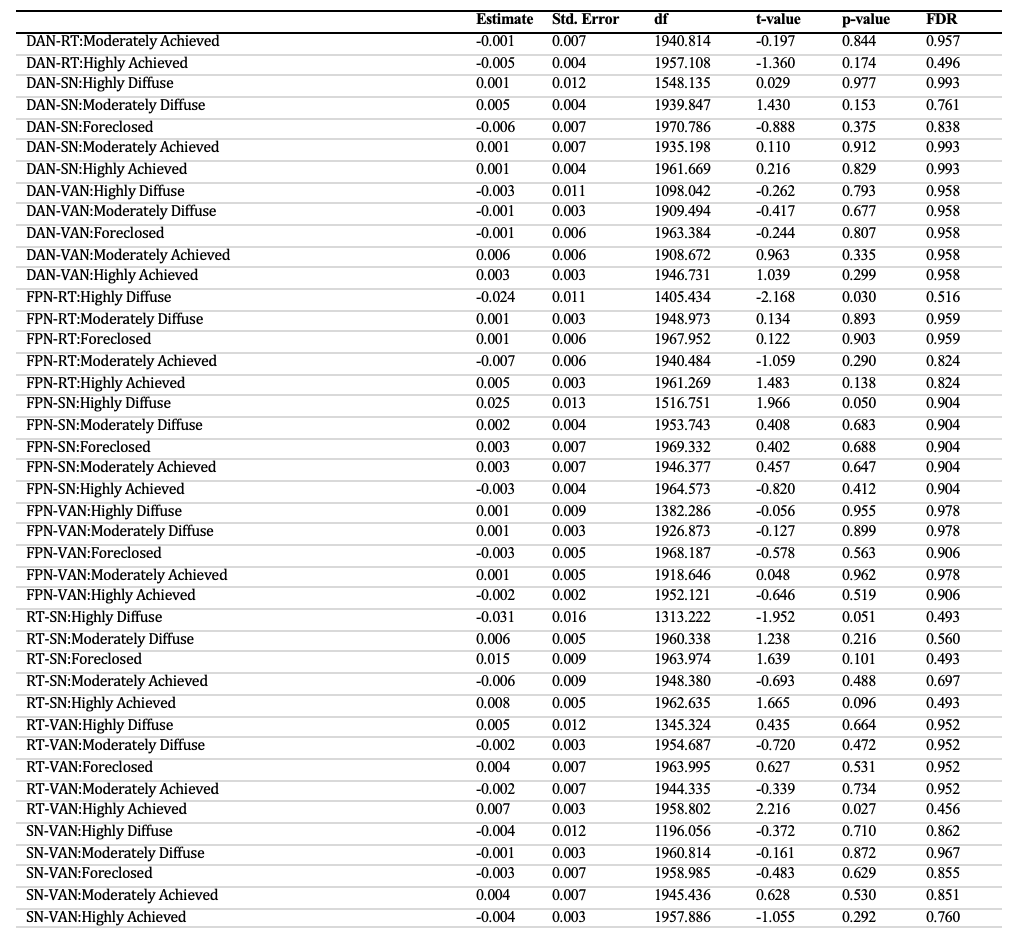
**

**Table S3. Moderation of Perceived Discrimination Results.** A moderation of perceived discrimination revealed within-CPAR significant ethnic identity profile associations. Statistical significance was determined by a p_FDR_–value < 0.05. Degrees of freedom are denoted as df, standard error is denoted as Std. Error, and false discovery rate is denoted as FDR.

**
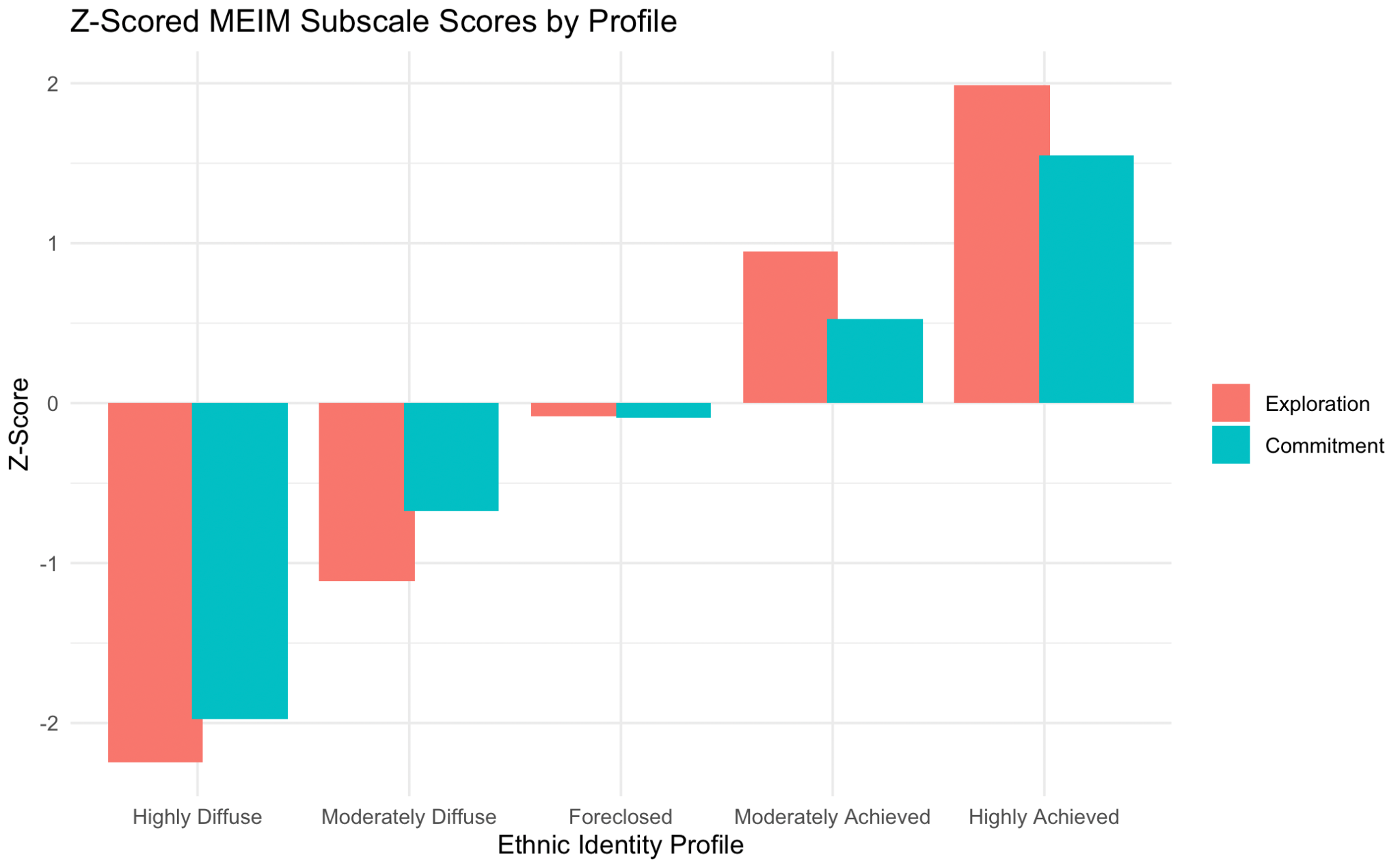
**

**Figure S1. Ethnic Identity Profiles by Z-Scores.**
